# Concurrent cytomegalovirus iridocyclitis and vitreoretinal lymphoma in the same eye during long-term infliximab therapy: a case report

**DOI:** 10.1186/s12348-026-00594-x

**Published:** 2026-05-14

**Authors:** Hitoshi Goto, Shiho Kunimatsu-Sanuki, Ryota Suga, Junko Hori

**Affiliations:** 1 Department of Ophthalmology, Nippon Medical School Tama-Nagayama Hospital, Tokyo, Japan; 2 Nishikasai Inouye Eye Hospital, Tokyo, Japan

**Keywords:** Cytomegalovirus iridocyclitis, Vitreoretinal lymphoma, Epstein–Barr virus, Infliximab, Immunosuppression, Opportunistic ocular infection

## Abstract

**Background:**

We report a rare case of cytomegalovirus (CMV) iridocyclitis and vitreoretinal lymphoma (VRL) that developed sequentially in the same eye during long-term infliximab (IFX) therapy for ulcerative colitis. This case highlights both the risk of opportunistic ocular infections and lymphoproliferative disorders coexisting in the same eye and the diagnostic challenges associated with prolonged immunosuppression.

**Case presentation:**

A 65-year-old man presented to the ocular inflammatory service at Nippon Medical School Tama-Nagayama Hospital with deteriorating vision in the left eye. His medical history included IFX therapy for 11 years for ulcerative colitis, a 7-year history of bilateral primary open-angle glaucoma, and recurrent iridocyclitis in the left eye. Slit-lamp examination revealed mutton-fat keratic precipitates and dense vitreous opacities. Multiplex PCR of the aqueous humor detected CMV and Epstein-Barr virus (EBV). The cytological grading of the vitreous fluid was class IIIb. Cytokine analysis revealed an interleukin (IL)-10/IL-6 ratio of > 1.0, and immunoglobulin heavy chain gene rearrangement revealed monoclonality. Based on these findings, the patient was diagnosed with concurrent CMV iridocyclitis and VRL in the same eye. EBV positivity in the aqueous humor may have been associated with VRL development under prolonged immunosuppression. Topical ganciclovir was initiated for CMV iridocyclitis. The patient underwent bilateral ocular radiotherapy (40 Gy) and systemic chemotherapy with rituximab, methotrexate, procarbazine, and vincristine after IFX cessation. Although inflammatory and infiltrative lesions resolved, his final visual acuity was 20/200 due to glaucomatous visual field loss.

**Conclusions:**

The present case highlights both the risk of opportunistic ocular infections and lymphoproliferative disorders associated with prolonged immunosuppression and the diagnostic challenge when both conditions coexist in the same eye. Careful monitoring and close collaboration between ophthalmologists and internists are essential for the early diagnosis and appropriate management of such patients.

## Introduction

Opportunistic infections, including herpetic keratouveitis [1] and endophthalmitis [2] have been reported to occur in patients during treatment with immunosuppressive agents such as infliximab (IFX); thus, careful attention by clinicians is warranted. Several cases of lymphoma have also been reported in patients receiving IFX [3, 4]. These lymphomas are classified as other iatrogenic immunodeficiency-associated lymphoproliferative disorders (OIIA-LPDs) [5]. Patients receiving immunosuppressive or biologic agents are therefore susceptible to both opportunistic ocular infections and lymphoproliferative disorders, highlighting the need for vigilant clinical monitoring [6, 7]. However, reports on cytomegalovirus iridocyclitis and malignant lymphoma in the same eye during immunosuppression are lacking. Herein, we report the case of a patient with ulcerative colitis who developed cytomegalovirus iridocyclitis and vitreoretinal lymphoma (VRL) in the same eye during long-term IFX therapy.

## Case presentation

**Fig. 1:**
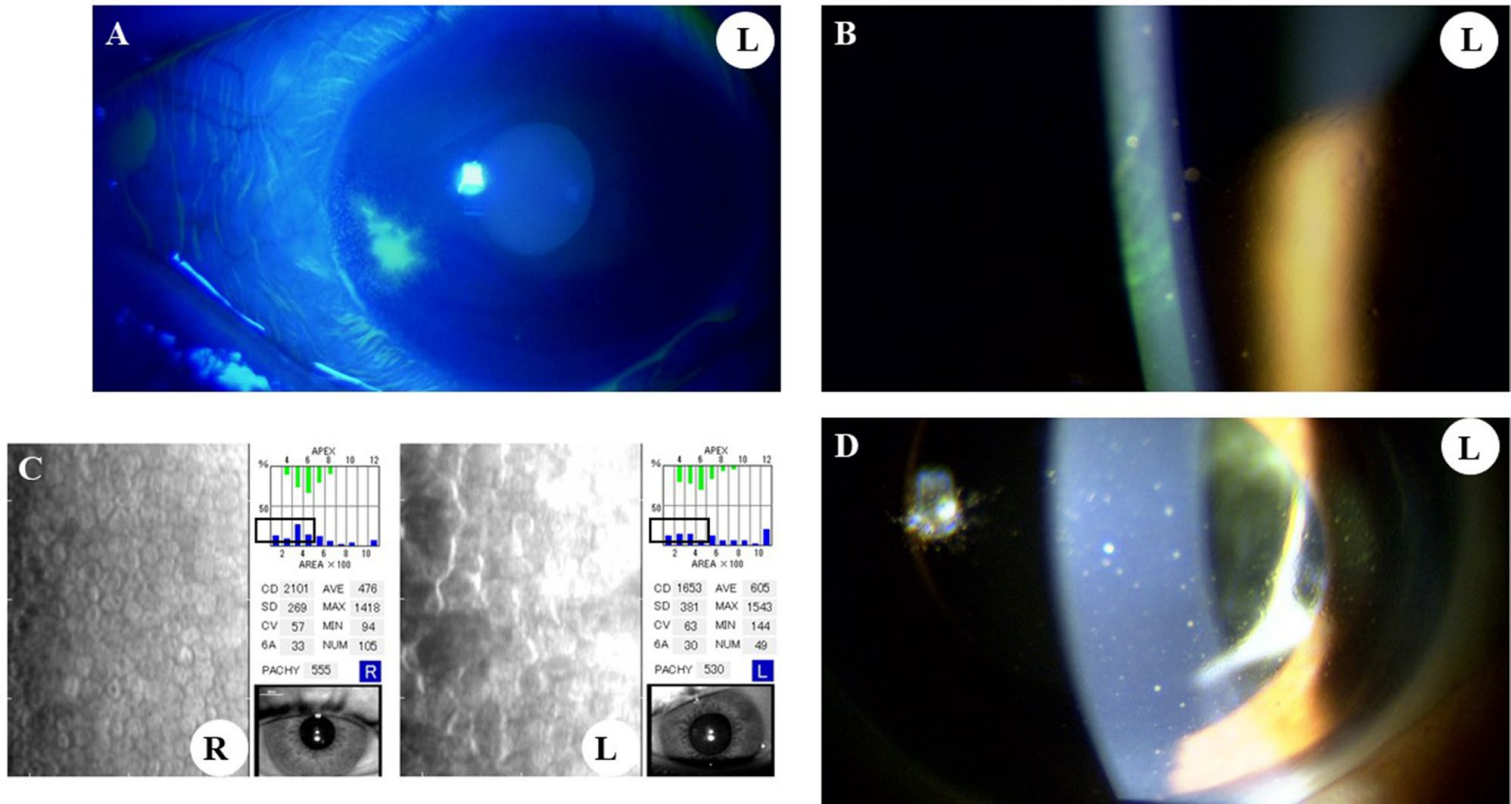
Clinical findings at the initial onset of dendritic keratitis and iridocyclitis at the referring hospital (**A–C**) and findings during the initial consultation at Nippon Medical School Tama-Nagayama Hospital (NMS-TN) 6 years and 10 months later (**D**). (**A**) Fluorescein staining of the left eye showing a dendritic corneal ulcer. (**B**) Slit-lamp examination of the left eye showing mutton-fat keratic precipitates distributed centrally and inferiorly. (**C**) Specular microscopy showing corneal endothelial cell densities of 2101 cells/mm² in the right eye and 1653 cells/mm² in the left eye, with a more pronounced decrease in the left eye. (**D**) Slit-lamp examination of the left eye at the initial presentation to NMS-TN, showing numerous mutton-fat keratic precipitates

A 65-year-old man presented to the ocular inflammatory service at Nippon Medical School Tama-Nagayama Hospital (NMS-TN) with deteriorating vision in the left eye at X (date of initial presentation to NMS-TN). At X − 7 years, the patient had been diagnosed with bilateral primary open-angle glaucoma at a referring hospital. Two months after the diagnosis (X − 6 years 10 months), the patient developed dendritic keratitis (Fig. 1A) and iridocyclitis with mutton-fat keratic precipitates distributed centrally and inferiorly (Fig. 1B) in the left eye for the first time. Specular microscopy revealed corneal endothelial cell densities of 2101 and 1653 cells/mm² in the right and left eye, respectively, with a more pronounced decrease in the left eye (Fig. 1C).

Topical acyclovir and oral valaciclovir were administered due to suspected herpetic inflammation. The recurrent iridocyclitis and elevated intraocular pressure in the left eye were subsequently managed as herpetic iridocyclitis-associated secondary glaucoma, although PCR testing of the ocular fluid was not performed at that time. Following the diagnosis of glaucoma, the patient underwent cataract surgery and trabeculotomy in the right eye and cataract surgery, trabeculotomy, and trabeculectomy with mitomycin C in the left eye. After trabeculectomy, his left intraocular pressure (IOP) was controlled at approximately 10 mmHg. Iridocyclitis recurred eight times in the left eye over the subsequent years, without accompanying keratitis, until referral to our hospital. The mean deviation on standard automated perimetry (Humphrey 24 − 2 SITA-Standard) was − 17.98 dB in the right eye and − 26.84 dB in the left eye.

At X − 3 months, iridocyclitis recurred in the left eye. The iridocyclitis partially improved with oral valaciclovir; however, vitreous opacity worsened. By X − 1 month, his vision deteriorated to counting fingers despite systemic corticosteroid therapy. Because these findings could not be fully explained by presumed herpetic iridocyclitis alone, vitreoretinal lymphoma (VRL) was suspected, and the patient was referred to NMS-TN.

**Fig. 2:**
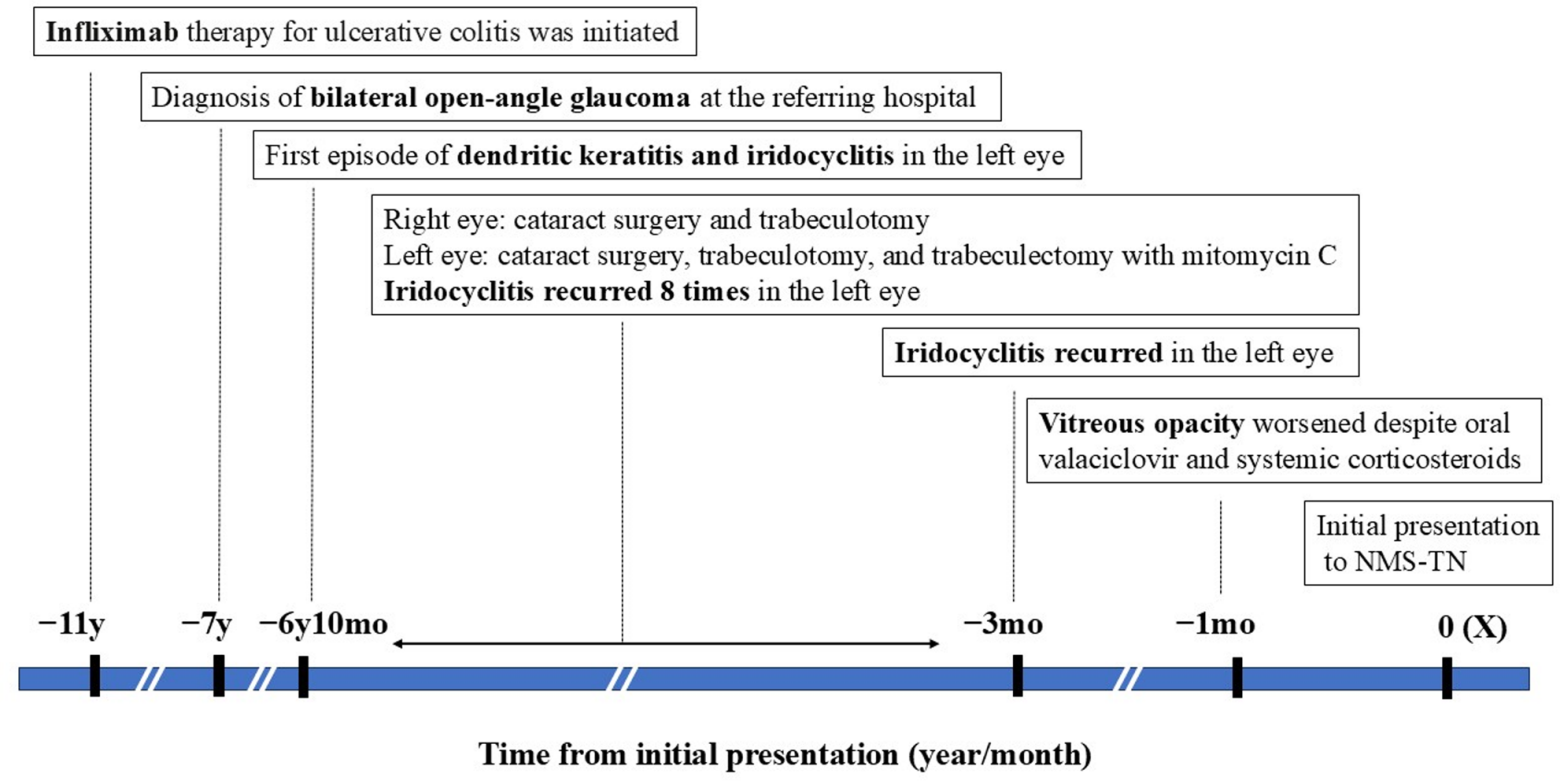
Chronology of the clinical history before referral to the Nippon Medical School Tama-Nagayama Hospital (NMS-TN). X denotes the date of the initial consultation at NMS-TN. Infliximab therapy for ulcerative colitis was initiated 11 years before the initial consultation (X − 11). At X − 7, the patient presented to the referring hospital for the first time, where clinicians diagnosed primary open-angle glaucoma in both eyes and initiated treatment. Two months later (X − 6 years 10 months), the patient developed keratitis and iridocyclitis in the left eye for the first time. Over the following years, cataract surgery and trabeculotomy were performed in both eyes, while trabeculectomy with mitomycin C was also performed in the left eye. During this period, iridocyclitis without keratitis recurred eight times in the left eye. At X − 3 months, iridocyclitis recurred in the left eye. At X − 1 month, the patient was referred to NMS-TN because vitreous opacities had worsened despite treatment with oral valaciclovir and systemic corticosteroids

The systemic medical history included polysplenia syndrome, hypertension, dyslipidemia, and ulcerative colitis diagnosed 12 years prior at the age of 53 years. Infliximab therapy was initiated 1 year after the diagnosis of ulcerative colitis (X − 11 years). The clinical course prior to referral is summarized in Fig. 2.

**Fig. 3:**
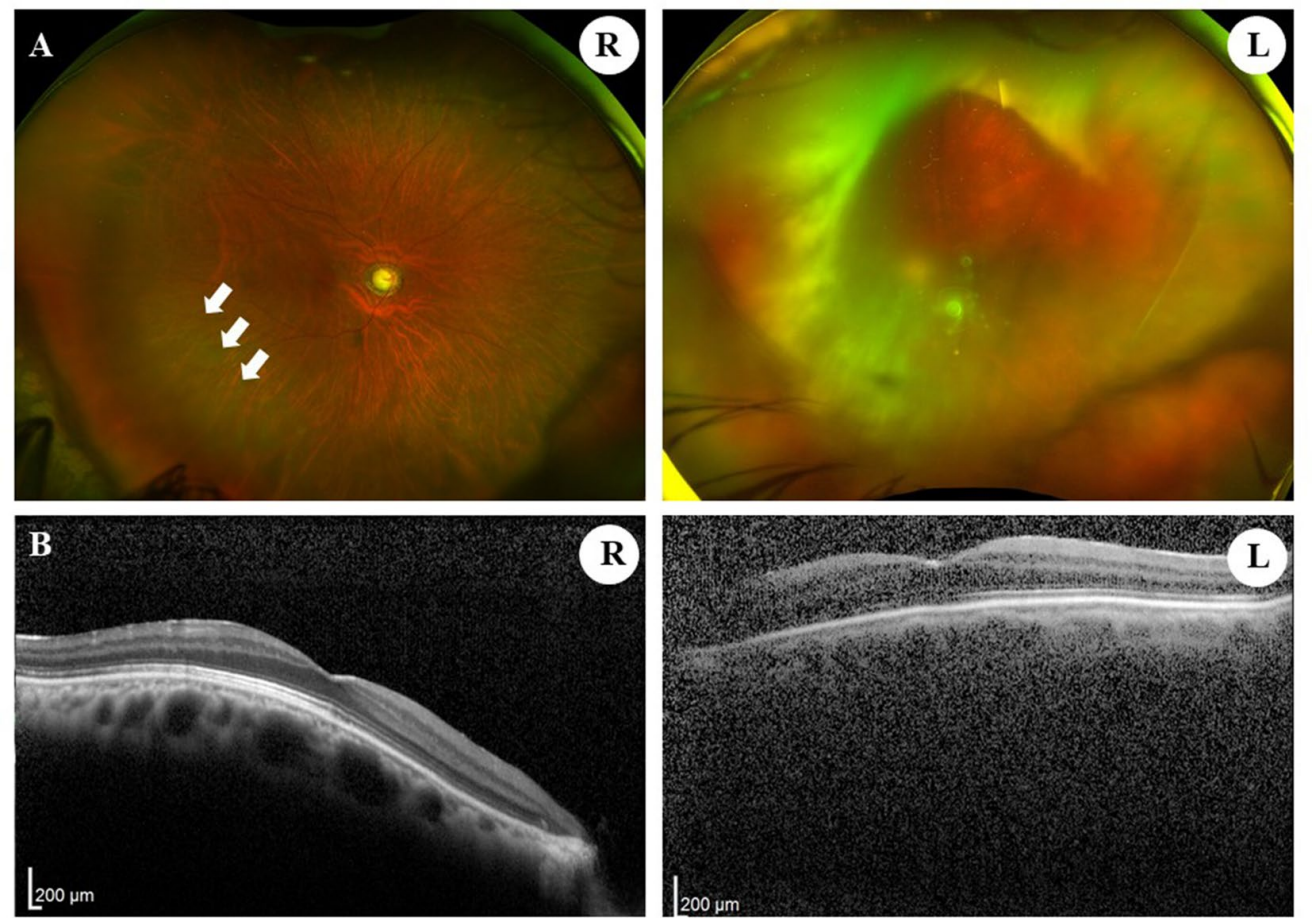
(**A)** Fundus examination revealed glaucomatous optic neuropathy in both eyes, with multiple whitish exudative lesions in the inferior retina of the right eye (arrow) and aurora-like vitreous opacities in the left eye. (**B)** Optical coherence tomography (OCT) showed no significant abnormalities in the right eye. In the left eye, visualization was limited due to vitreous opacities, but no evident abnormalities were detected within the visible area

At the initial visit (X), his best corrected visual acuity was 20/20 in the right eye and hand motion in the left eye. Intraocular pressure was 10 and 13 mmHg in the right and left eye, respectively. Slit-lamp examination of the left eye revealed numerous mutton-fat keratic precipitates (Fig. 1D), 2 + anterior chamber cells, and 3 + vitreous cells. Only trace anterior chamber cells were detected in the right eye. Both eyes were pseudophakic. Fundus examination revealed glaucomatous optic neuropathy in both eyes, multiple whitish exudative lesions in the inferior retina of the right eye, and aurora-like vitreous opacities in the left eye (Fig. 3A). Optical coherence tomography revealed no significant abnormalities in the right eye. Visualization of the posterior segment of the left eye was limited due to vitreous opacities; however, no abnormalities were detected in the visible area (Fig. 3B). 

Specular microscopy revealed reduced corneal endothelial cell densities of 1866 and 1297 cells/mm² in the right and left eye, respectively; the decrease was more pronounced in the left eye. Blood tests revealed no significant abnormalities.

At presentation, the patient was receiving mesalazine (4800 mg/d) for ulcerative colitis. Oral prednisolone (15 mg/d, tapered from 30 mg/d) and oral valaciclovir (500 mg/d, tapered from 1000 mg/d) were also prescribed at the referring hospital. Topical medications for the left eye included betamethasone (four times daily), tropicamide/phenylephrine (once daily), and acyclovir ointment (thrice daily). Latanoprost/timolol and brinzolamide were also administered in both eyes for glaucoma management. Diagnostic vitrectomy of the left eye was conducted 3 d after presentation. Aqueous humor sampling and vitreous biopsy were performed simultaneously. At the start of the surgery, aqueous humor (approximately 0.4 mL) was aspirated, followed by collection of undiluted vitreous fluid (approximately 1 mL) and standard vitrectomy before initiating intraocular infusion. Diluted vitreous irrigation fluid aspirated during surgery was also collected at the end of the procedure.

**Fig. 4:**
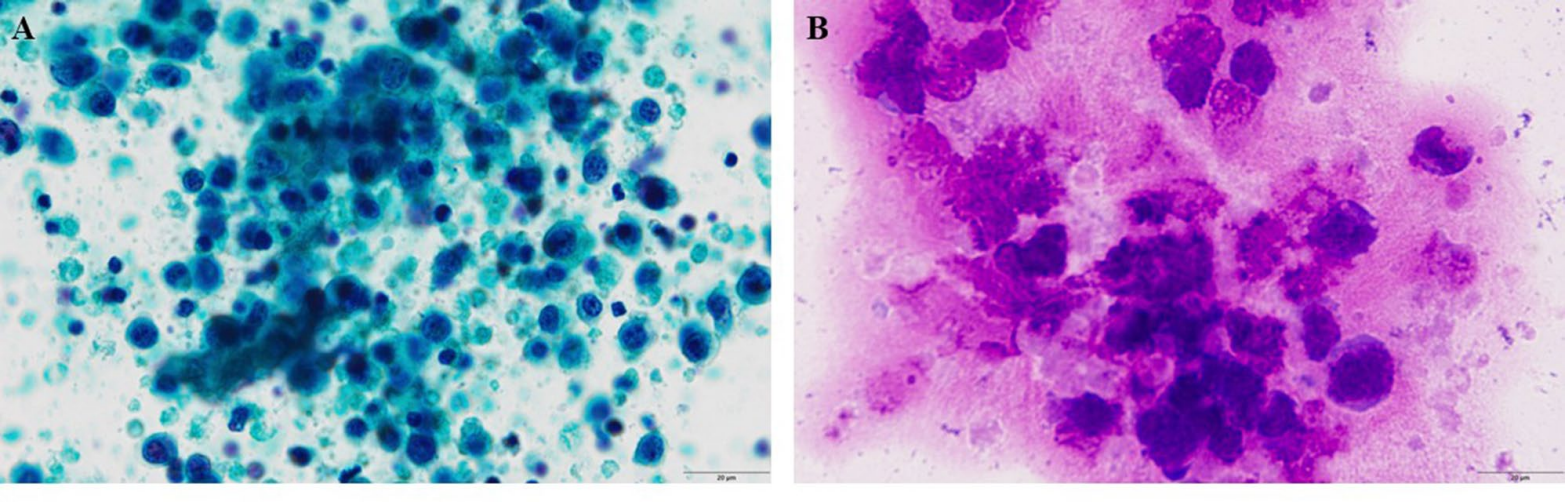
Cytologic examination of the vitreous fluid from the left eye. (**A)** Papanicolaou staining (objective lens ×60). (**B)** Giemsa staining (objective lens ×60). Irregular nuclei with marked anisokaryosis and multinucleated giant cells are observed

**Fig. 5:**
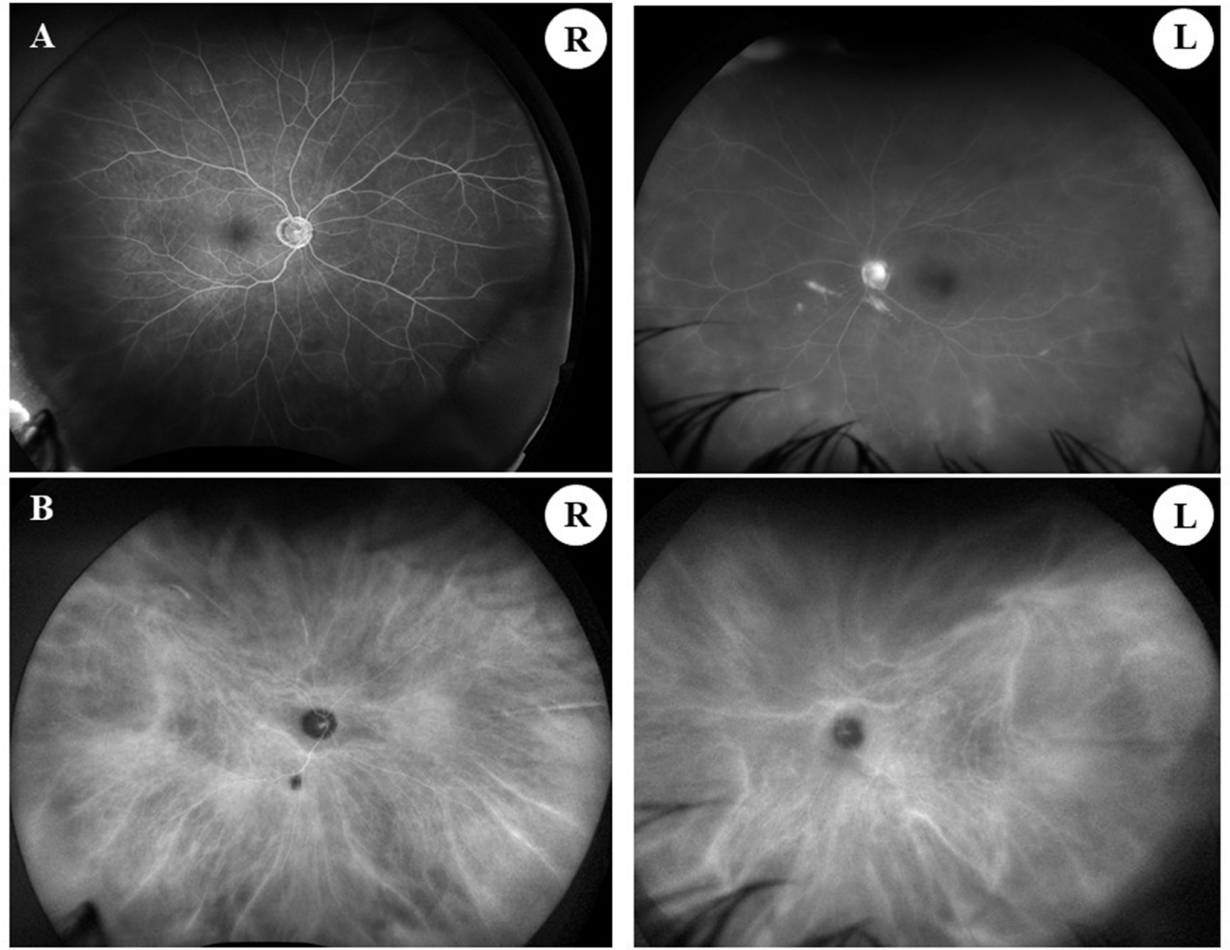
(**A)** Fluorescein angiography of the left eye showed dye leakage from the retinal veins. **(B)** Indocyanine green angiography did not show any blockage suggesting subretinal infiltration

Comprehensive PCR testing of the aqueous humor detected cytomegalovirus (CMV) and Epstein–Barr virus (EBV) but not herpes simplex virus types 1 and 2, or varicella-zoster virus.　 Cytological examination of the vitreous samples revealed class IIIb findings (Fig. 4A–B).　Cytokine analysis revealed increased interleukin (IL)-10 (91 pg/mL) and IL-6 (51.2 pg/mL) levels, with an IL-10/IL-6 ratio of > 1.0. PCR analysis of immunoglobulin heavy chain gene rearrangement revealed monoclonality in three regions. Based on these findings, the patient was diagnosed with CMV iridocyclitis and VRL. Flow cytometric analysis could not be performed because of the insufficient number of cells. Analysis of the L265P mutation in the myeloid differentiation primary response 88 gene (MYD88) was not performed. EBV-encoded small RNA in situ hybridization (EBER-ISH) of the vitreous specimen was also not performed. Magnetic resonance imaging of the brain and orbits with and without contrast did not show evidence of central nervous system lymphoma. Fluorine-18 fluorodeoxyglucose positron emission tomography/computed tomography (FDG-PET/CT) of the chest, abdomen, and pelvis did not show evidence of local or metastatic malignancy. Postoperatively, fluorescein angiography of the left eye revealed dye leakage from the retinal veins after vitrectomy (Fig. 5A). Indocyanine green angiography did not reveal any blockage suggestive of subretinal infiltration (Fig. 5B). 

**Fig. 6:**
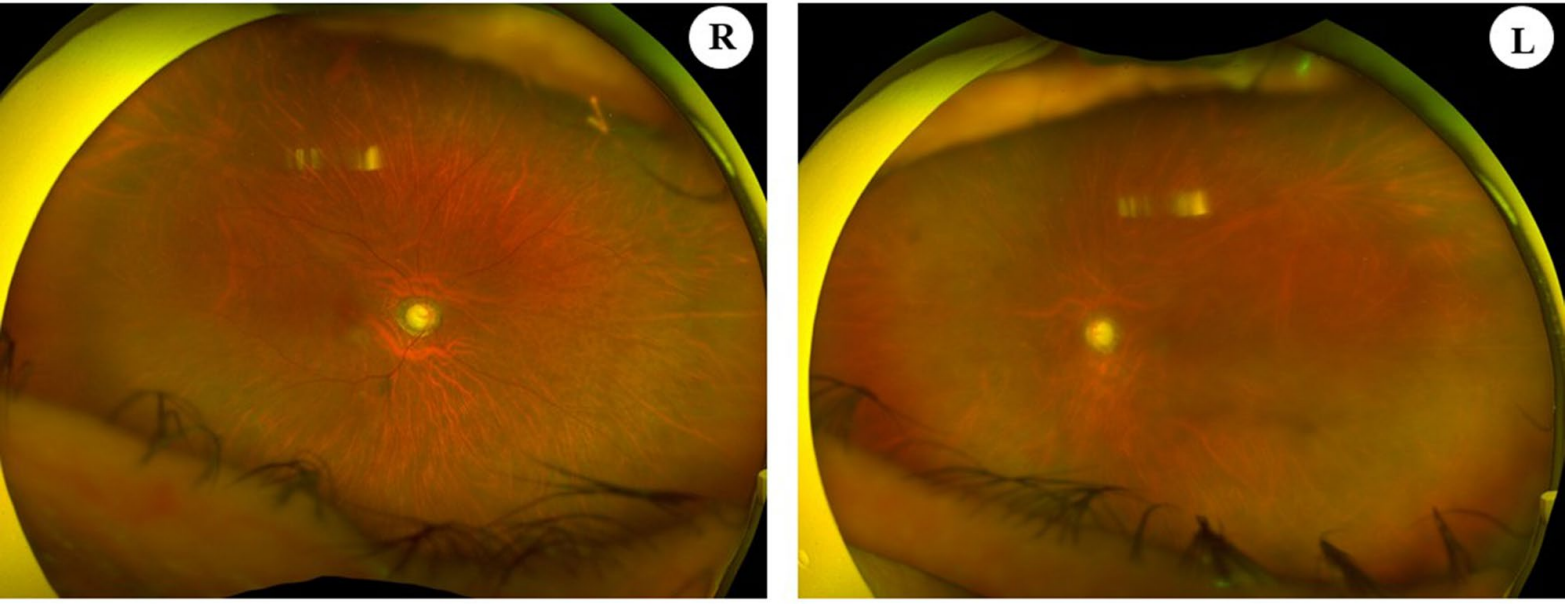
The vitreous opacities and exudative lesions in both eyes remained resolved at five months after the initial visit

Oral prednisolone, which had been initiated at the referring hospital, was tapered by 5 mg every 5 d postoperatively and discontinued over approximately 3 weeks. Oral valaciclovir (500 mg/d) was also discontinued 5 d after vitreous surgery. IFX therapy for ulcerative colitis was discontinued in collaboration with the hematology department, with no exacerbation of ulcerative colitis confirmed by a gastroenterologist. Topical ganciclovir (2%) was initiated on postoperative day 5 for the treatment of CMV iridocyclitis. Bilateral ocular radiotherapy (40 Gy) was started 1 month after surgery and completed over the following month. Following completion of radiotherapy, systemic chemotherapy with rituximab, methotrexate, procarbazine, and vincristine (R-MPV) was administered every 2 weeks for five cycles. The vitreous opacities and exudative lesions in both eyes gradually improved with treatment, and the visual acuity of the left eye improved to 20/70 at 1 month after the initial visit. However, the patient developed radiation keratopathy, and visual acuity decreased to 20/200. By 5 months after the initial visit, keratopathy improved and the vitreous opacities and exudative lesions in both eyes resolved (Fig. 6).

The final visual acuity of the left eye was 20/200 due to central visual field loss from glaucoma.

## Discussion

The present case highlights two important points. First, opportunistic infections and malignant diseases may occur during treatment with immunosuppressive drugs such as IFX, regardless of treatment duration. Second, long-term immunosuppression may lead to the coexistence of opportunistic ocular infections and malignant tumors in the same eye. Several opportunistic ocular infections have been reported in patients with immunosuppression. Herpetic keratouveitis was diagnosed in a patient with Behçet’s disease 6 months after IFX initiation [1]. Endophthalmitis was diagnosed in another patient during IFX treatment for surgically induced necrotizing scleritis [2]. Antitumor necrosis factor-α (TNF-α) agents and other biologic therapies have been associated with an increased risk of opportunistic ocular infections [6, 7].

In the present case, CMV iridocyclitis was diagnosed based on anterior chamber fluid PCR findings after 11 years of IFX therapy for ulcerative colitis. CMV infects the trabecular meshwork and corneal endothelium, and reactivation of the latent virus during immunosuppression is thought to cause recurrent inflammation [8–11]. The initial episode of dendritic keratitis and iridocyclitis in the left eye likely led to a clinical diagnosis of herpetic uveokeratitis at the referring hospital. However, because PCR testing of the ocular fluid was not performed at that time, whether the subsequent recurrent iridocyclitis was herpetic or CMV-related remains uncertain. In addition, ulcerative colitis had remained clinically stable since the initiation of infliximab therapy 11 years earlier, making ulcerative colitis-associated iridocyclitis less likely. Eventually, the diagnosis of CMV iridocyclitis was confirmed only after PCR testing at our hospital. Multiplex PCR (Strip PCR) for 24 ocular pathogens has recently become available in Japan and is useful for diagnosing CMV iridocyclitis [10]. However, insurance coverage is not yet available. Although iridocyclitis itself does not entirely exclude a masquerade syndrome [12], the positive PCR result for CMV in the aqueous humor, combined with the long-standing history of recurrent iridocyclitis and elevated intraocular pressure, supported a concurrent diagnosis of CMV iridocyclitis. Miyazaki et al. reported a sensitivity of 90.0% and specificity of 98.7% for quantitative real-time PCR in diagnosing ocular CMV infection, thereby providing strong support for the validity of a positive result [13]. The coexistence of CMV iridocyclitis and VRL in the same eye, although uncommon, appeared plausible in the present case. Moreover, delayed diagnosis of CMV likely led to frequent recurrences of iridocyclitis and ocular hypertension, resulting in worsening glaucoma, which contributed to the final vision loss. Systemic ganciclovir represents a potential treatment option for CMV iridocyclitis [14, 15]; however, it may be associated with serious adverse effects, including pancytopenia and renal dysfunction [15]. Therefore, topical ganciclovir was initially selected because it has fewer systemic adverse effects and allows safer long-term use. In the present case, iridocyclitis did not recur between the completion of surgery and the initiation of radiotherapy despite the discontinuation of systemic corticosteroids, suggesting that topical ganciclovir effectively controlled CMV iridocyclitis during this interval.

Malignancies may also occur during immunosuppression. Sonoda et al. reported a case of diffuse large B-cell lymphoma (DLBCL) after 4 years of IFX therapy for Behçet’s disease [3]. Furusawa et al. reported a case of DLBCL and peripheral T-cell lymphoma not otherwise specified (PTCL-NOS) in a patient with sarcoidosis and ankylosing spondylitis after 9 years of IFX therapy [4]. These cases are classified as OIIA-LPD and are associated with EBV infection, underlying autoimmune disease, and immunosuppressive therapy [5]. In the present case, the patient had received IFX therapy for ulcerative colitis for 11 years. Despite oral valaciclovir administration and systemic corticosteroid therapy for recurrent iridocyclitis in the left eye, vitreous opacity progressively worsened, leading to suspicion of VRL in the context of other iatrogenic immunodeficiency-associated lymphoproliferative disorder (OIIA-LPD).

TNF-α plays a critical role in immune surveillance; its inhibition impairs natural killer cell activation and reduces cytotoxic activity against EBV-infected B-cells [16]. Under sustained immunosuppression, EBV may escape immune control, potentially driving B-cell lymphoproliferation and contributing to lymphomagenesis [17]. EBV positivity has been reported in approximately 50–70% of OIIA-LPD cases. In the present case, EBV was detected in the aqueous humor via PCR, suggesting a possible association with VRL development in the context of IFX-induced immunosuppression. However, PCR-based detection of EBV DNA cannot distinguish between active infection and latent viral presence, and EBV DNA may be detected even in healthy individuals; therefore, careful interpretation of a positive result is required [18]. EBV-positive large B-cell lymphoma with vitreoretinal involvement (LBCL-VR) has recently been recognized as a distinct entity from conventional LBCL-VR, characterized by CD30 positivity and the absence of MYD88 and CD79A mutations, which are mutually exclusive with EBV positivity [17]. Although MYD88 mutation testing is a valuable diagnostic tool for VRL, it has not yet been incorporated as a mandatory diagnostic criterion in Japan. VRL was diagnosed on the basis of cytopathological findings, immunoglobulin heavy-chain (IgH) gene rearrangement, and an IL-10/IL-6 ratio exceeding 1 [19]. However, MYD88 mutation testing, CD30 expression analysis, and EBER-ISH were not performed; therefore, definitive subclassification as EBV-positive LBCL-VR could not be performed. Notably, Zafar et al. reported a case of EBV-positive LBCL-VR in which EBER-ISH confirmed EBV positivity in atypical lymphocytes, with no evidence of MYD88 L265P mutation [17]. The clinicopathological features of EBV-positive LBCL-VR remain incompletely defined and may overlap with lymphoproliferative disorders arising in the setting of iatrogenic immunodeficiency, including OIIA-LPD.

Although some OIIA-LPDs, especially methotrexate-associated lympho**p**roliferative disorder (MTX-LPD), regress after discontinuation of immunosuppressive drugs (ISDs) [20], such an approach may not be feasible in ophthalmology because visual function is highly time-sensitive. Hamano et al. [21] reported a patient with rheumatoid arthritis receiving MTX for > 20 years who developed CMV retinitis and VRL. Despite MTX cessation and combined local and systemic treatment, visual recovery was limited. Previous reviews have reported a wide range of spontaneous regression rates of lymphoproliferative disorders after immunosuppressive drug discontinuation, ranging from approximately 20% to 70% [20]. However, given the time-sensitive nature of visual function, immunosuppressive discontinuation alone may be insufficient in ocular cases, and early initiation of treatment may be required. Although no standard treatment has been established for VRL, the 2024 clinical practice guidelines of the Japan Society for Neuro-Oncology recommend ocular or whole-eye and brain radiotherapy as the initial treatment from the perspective of insurance coverage in Japan [22]. Intravitreal MTX injection also represents a treatment option for intraocular lesions; however, it is not covered by insurance in Japan. VRL frequently involves the central nervous system, with brain involvement reported in 65–80% of cases either at diagnosis or within 29 months after diagnosis [23], and is associated with a poor prognosis. Therefore, treatment strategies that include systemic chemotherapy in addition to local therapy have been considered to prevent central nervous system dissemination [24]. For these reasons, local treatment alone was considered to be insufficient in the present case because of the risk of central nervous system involvement. After consultation with hematologists, the patient was treated with bilateral ocular radiotherapy in combination with systemic chemotherapy, as exudative lesions were observed in both eyes at presentation [25].

In summary, long-term treatment with IFX can lead to opportunistic infections and malignant lymphoproliferative disease in the same eye. The sequential occurrence of CMV iridocyclitis and VRL during prolonged immunosuppression is extremely rare. These conditions may develop at different time points or coexist, complicating the diagnostic process. The present case highlights the need for ophthalmologists and internists to remain vigilant for infections and malignancies during immunosuppressive therapy, regardless of its duration. Clinicians should collaborate closely to adjust ISDs and systemic and local treatments to preserve visual function.

## Data Availability

All data generated or analyzed during this study are included in this published article. Further inquiries can be directed to the corresponding author.

## Abbreviations

CMV: Cytomegalovirus
DLBCL: Diffuse large B-cell lymphoma
EBV: Epstein-Barr virus
EBER-ISH: EBV-encoded small RNA in situ hybridization
FDG-PET/CT: Fluorine-18 fluorodeoxyglucose positron emission tomography/computed tomography
ISDs: Immunosuppressive drugs
IFX: Infliximab
IL: Interleukin
IOP: Intraocular pressure
LBCL-VR: Large B-cell lymphoma with vitreoretinal involvement
MTX-LPD: Methotrexate-associated lymphoproliferative disorder
MYD88: Myeloid differentiation primary response 88
OIIA-LPDs: Other iatrogenic immunodeficiency-associated lymphoproliferative disorders
PTCL-NOS: Peripheral T-cell lymphoma not otherwise specified
R-MPV: Rituximab, methotrexate, procarbazine, and vincristine
TNF-α: Tumor necrosis factor-alpha
VRL: Vitreoretinal lymphoma
